# Sex differences and acute intermittent isometric handgrip exercise effects on microvascular reactivity in young healthy adults

**DOI:** 10.3389/fphys.2026.1895045

**Published:** 2026-10-13

**Authors:** Wonjae Lee, Elizabeth G. Knoll, Kase J. Pennartz, Justin D. Sprick, Joshua L. Keller

**Affiliations:** Applied Physiology Laboratory, Department of Kinesiology, Health Promotion and Recreation, School of Health Sciences, College of Health and Public Service, University of North Texas, Denton, TX, United States

**Keywords:** cardiovascular physiology, microcirculation, near infrared spectroscopy, peripheral vascular function, reactive hyperemia, skeletal muscle metabolism

## Abstract

**Introduction:**

Near-infrared spectroscopy combined with a vascular occlusion test (NIRS-VOT) is a popular approach for investigating microvascular function across many settings. Although sex differences and exercise-induced changes in NIRS-VOT responses have been reported, limited evidence exists detailing whether these responses persist after accounting for confounding factors such as adipose tissue thickness (ATT) and the magnitude of the ischemic stimulus. Therefore, the purpose of this study was twofold: 1) to determine whether sex explains variability in NIRS-VOT outcomes beyond that accounted for by ATT and measures of the ischemic stimulus, and 2) to determine the extent to which acute isometric handgrip exercise influences NIRS-VOT responses in a sex-specific manner.

**Methods:**

Twenty-four healthy adults (12 males) completed two experimental visits consisting of repeated NIRS-VOTs performed before and after either rhythmic handgrip exercise (30 contractions at 50% maximal voluntary contraction) or time-matched rest. Ischemia was terminated once 40% skeletal muscle tissue oxygenation (StO_2_) was observed to standardize the ischemic stimulus. Model comparison analyses evaluated the predictive contributions of sex, ATT, and cumulative O_2_ deficit to upslope, maximal saturation (StO_2max_), and half-time to StO_2max_ (T_1/2_). Linear mixed-effects models examined the effects of Sex, Condition, and Time on NIRS-VOT outcomes.

**Results:**

Sex was the strongest predictor of upslope, explaining approximately 15% of the variance, whereas cumulative O_2_ deficit was the strongest predictor of StO_2max_ and T_1/2_. Acute exercise increased downslope and upslope while reducing StO_2max_ (all p < 0.05). However, no Sex × Condition × Time interactions were detected (all p > 0.05). Because the study was not powered to detect higher-order interactions, the sex-specific exercise analyses should be considered exploratory rather than evidence that males and females respond equivalently.

**Discussion:**

Overall, these results demonstrate that determinants of NIRS-VOT responses are outcome-specific and influenced by both skeletal muscle metabolic characteristics and microvascular function. Cumulative O_2_ deficit emerged as a primary determinant of StO_2max_, supporting a muscle-centric framework for interpreting at least some NIRS-derived measures of reactive hyperemia.

## 1 Introduction

Near-infrared spectroscopy (NIRS) has gained popularity as a non-invasive tool for assessing skeletal muscle tissue oxygenation (StO_2_, %) across clinical, athletic, and recreational populations (Keller and Kennedy, 2021; Rasica et al., 2022; Zaleski et al., 2022; Keller et al., 2023; Rogers et al., 2023; Alvares et al., 2024; Citherlet et al., 2024; Landers-Ramos et al., 2024; Perlet et al., 2024; Traylor et al., 2024; Chatlaong et al., 2026; Landers-Ramos et al., 2026; Murias et al., 2026). One common application involves evaluating changes in StO_2_ during a vascular occlusion test (VOT), which provides insight into microvascular function and, in previous studies, has been associated with cardiovascular disease (CVD) risk factors such as hypertension, inflammation, and endothelial dysfunction (de Oliveira et al., 2019; Alvares et al., 2024). A standard VOT protocol consists of a period of transient ischemia followed by reperfusion, during which various NIRS-derived resaturation indices are calculated to characterize the hyperemic response. Although NIRS-VOT does not directly measure blood flow, researchers commonly use tissue oxygenation kinetics during reperfusion to characterize NIRS-derived indices of microvascular reactivity. The magnitude of this response has been identified as a potent predictor of CVD risk (Anderson et al., 2011), with evidence suggesting notable sex differences in the mechanisms underlying this risk (Stanhewicz et al., 2018; Robinson et al., 2019), emphasizing the need to identify the precise physiological contributors to these resaturation indices. This response, reactive hyperemia, is mediated by a combination of myogenic, metabolic, and endothelial factors within the microvasculature (Crecelius et al., 2013a; 2013b; Rosenberry and Nelson, 2020). With respect to NIRS-derived outcomes, individual resaturation variables may reflect different underlying physiological processes. Common indices include the initial rate of reoxygenation (upslope), peak oxygenation (StO_2max_), and time to peak (or half time to peak; T_1/2_). Of note, these variables do not exhibit uniform relationships with known drivers of reactive hyperemia (Rasica et al., 2022; Keller et al., 2023; Traylor et al., 2024). For example, we, as well as others, previously reported that the magnitude of hypoxia experienced during the ischemic phase (i.e., StO_2min_; termed the ischemic vasodilatory stimulus) was strongly related to upslope, whereas no such relation was observed with StO_2max_ (Rasica et al., 2022; Keller et al., 2023). Although both are used to characterize the same reperfusion phase, these findings suggest they are governed by distinct physiological factors. Identifying which indices are most closely linked to specific metabolic, myogenic, and/or endothelial contributions may improve the utility of NIRS-VOT for detecting early vascular dysfunction and monitoring responses to interventions such as exercise or dietary modification.

To further delineate the factors influencing resaturation variables (upslope, StO_2max_, and T_1/2_), examining their change in response to a non-fatiguing exercise bout provides a practical experimental approach, particularly when controlling for the magnitude of the ischemic vasodilatory stimulus (i.e., StO_2min_) (Rosenberry et al., 2019; Keller et al., 2023). Specifically, a non-fatiguing protocol enables examination of acute metabolic perturbations while minimizing confounding influences from fatigue-related mechanisms, including task failure (e.g., motivation) and known sex differences in fatigue resistance (Hunter, 2016; Pennartz et al., 2026). It is well established that exercise increases muscle oxygen consumption (mV̇O_2_) and the production of metabolic byproducts (e.g., adenosine and K^+^); therefore, VOT outcomes obtained immediately post-exercise are expected to be influenced by the elevated metabolic state. To reduce confounding effects, anchoring the occlusion duration to a target StO_2min_, rather than a fixed 5-minute interval, allows standardization of the ischemic vasodilatory stimulus across conditions and participants (Rosenberry et al., 2019). This approach (when combined with supported covariates) can better characterize physiological factors that influence NIRS-derived responses, especially when ischemic duration is also considered (Alvares and Soares, 2024; Murias et al., 2026). It also remains important to evaluate these responses in a sex-specific context, as accumulating evidence, including our past findings, indicates sex differences in microvascular function exist.

In healthy young adults, NIRS-VOT provides a useful approach for examining physiological variability in skeletal muscle characteristics and microvascular reactivity. Establishing how biological sex, adipose tissue, and skeletal muscle metabolic factors influence these outcomes in healthy adults is also important before extending NIRS-VOT measurements to older or clinical populations. Thus, within this population, we have completed a series of investigations demonstrating that men desaturate and resaturate more rapidly and to a greater overall magnitude in response to a VOT than women (Keller and Kennedy, 2021; Keller et al., 2023; Perlet et al., 2024; Traylor et al., 2024). These studies have suggested that, even when StO_2min_ is experimentally controlled, men exhibit greater reactive hyperemia (as measured by NIRS) (Keller et al., 2023), and that increasing limb blood flow via passive heating does not elicit comparable resaturation responses in women relative to men under non-heated conditions (Traylor et al., 2024). Several independent lines of research have largely corroborated and extended these findings (Rasica et al., 2022; Zaleski et al., 2022; Citherlet et al., 2024; Chatlaong et al., 2026; Murias et al., 2026). To be clear, however, not all laboratory groups report sex-related differences in NIRS-derived microvascular function (Landers-Ramos et al., 2024; Landers-Ramos et al., 2026). For example, although men often exhibit a greater StO_2_ range and steeper desaturation and resaturation slopes than women, these differences may be attenuated when confounding factors such as adipose tissue thickness (ATT) are accounted for. Landers-Ramos et al. (2024) reported that applying a regression-based correction for ATT attenuated sex differences in the resaturation slope; however, this approach relies on a pooled ATT–outcome relationship and may obscure independent or sex-specific effects that would be more rigorously evaluated using multivariable or model comparison approaches (Chatlaong et al., 2026). Accordingly, we acknowledge that biological sex may exert only a modest influence on desaturation indices, with other factors (e.g., ATT and age) likely contributing more (Perlet et al., 2024). In contrast, we maintain that biological sex remains a significant and meaningful predictor of various hyperemic responses (Keller et al., 2023; Murias et al., 2026). In support of this, our past work demonstrated that for StO_2max_ sex accounted for ~26% of the variance and exhibited substantially greater explanatory power than ATT or the ischemic vasodilatory stimulus (R^2^ = ~0), with stronger model evidence that sex was the dominant predictor (Bayes Factor = 90 – 100) (Keller et al., 2023), which may be due to differences intrinsic to the skeletal muscle like size and fiber type proportions. It remains unknown, however, whether these differences persist under conditions of heightened metabolic stress (e.g., post-exercise), or whether post-exercise responses in women approximate pre-exercise responses in men. Addressing this gap may help clarify the extent to which metabolic factors contribute to sex differences recorded during reperfusion. Furthermore, examining pre-exercise responses within a unified framework that incorporates StO_2min_, ATT, ischemic duration, and biological sex, using both experimental and statistical controls, may provide meaningful mechanistic insight. To date, however, no study has comprehensively evaluated these factors, particularly in the context of metabolic perturbation.

Therefore, the aims of this study were twofold: 1) to determine whether sex explains variability in NIRS-VOT outcomes beyond that accounted for by ATT and measures of the ischemic stimulus, and 2) to explore the extent to which acute isometric handgrip exercise influences NIRS-VOT responses, particularly in a sex-specific manner. Based on previous investigations (Keller et al., 2023; Landers-Ramos et al., 2024; Landers-Ramos et al., 2026; Murias et al., 2026), it was hypothesized that men would exhibit higher overall NIRS-VOT metrics than women. Additionally, it was hypothesized that exercise would augment these responses in a sex-specific manner when the magnitude of the ischemic vasodilatory stimulus (i.e., StO_2min_) was experimentally controlled. It was important to test these hypotheses, as clarifying the physiological and methodological basis of sex differences in microvascular function may provide insight into mechanisms underlying sex-specific cardiovascular risk and further inform the interpretation of non-invasive vascular assessments.

## 2 Materials and methods

### 2.1 Ethics statement

The current investigation was not preregistered in any database and was approved by the University of North Texas Institutional Review Board for Human Subjects research (IRB-25-541). Additionally, an IRB amendment/modification was submitted and approved by the local IRB to include a control condition to examine the effect of repeated VOTs on NIRS metrics on March 6, 2026. All procedures were conducted in accordance with the ethical standards established by the Declaration of Helsinki 2013. Written Informed Consent, Physical Activity Readiness Questionnaire (PARQ+), and a health history questionnaire were obtained from all participants prior to enrollment in the investigation.

### 2.2 Experimental design

To address the purpose of the current investigation, a mixed, repeated-measures experimental design was used to examine sex differences in NIRS-VOT responses following two conditions: a non-fatiguing handgrip exercise (EX) and a non-exercise control (CON). Participants completed two laboratory visits. Because the CON condition was added through an IRB amendment after participant enrollment had begun, the order of the conditions was not randomized. Rather, all participants completed the EX condition during their first laboratory visit, followed by the CON condition visit. During each visit, a baseline VOT (PRE) was performed, followed by either a bout of non-fatiguing intermittent handgrips (50% maximal effort) or 5 min of seated rest (CON), after which a second VOT was conducted (POST). Thus, NIRS-VOT metrics were assessed pre- and post-condition within each visit to evaluate the acute effects of exercise versus rest, with biological sex as the between-subjects factor. Ultrasonography and body composition were also performed to collect participant characteristics, including subcutaneous ATT, height, weight, lean body mass, and percent body fat, as displayed in **Table 1**.

**Table 1:** Participant characteristics by sex.

Characteristic	Males	Females	p-value	Mean difference (95% CI)
Age (yr)	26 ± 5	22 ± 4	0.090	-0.5 to 7.2
Weight (kg)	88.3 ± 18.3	61.9 ± 8.6	<0.001	15.4 to 37.9
Height (cm)	176.2 ± 11.7	163.0 ± 7.9	0.002	5.4 to 22.2
Body mass index (kg·m^-2^)	28.3 ± 3.9	23.4 ± 3.3	<0.001	2.3 to 8.7
Body fat (%)	22.3 ± 7.9	28.4 ± 6.6	0.051	-11.1 to 0.1
Adipose tissue thickness (cm)	0.377 ± 0.095	0.460 ± 0.105	0.054	-0.16 to 0.00

Values are mean ± SD; n = 12 per sex. 95% confidence interval (CI) of the mean difference between groups was calculated as males − females. BMI, body mass index; ATT, adipose tissue thickness.

### 2.3 Human participants

A total of 28 participants were enrolled in this study; however, four did not complete both visits due to personal and/or scheduling-related reasons. Thus, 24 healthy adults (12 males) completed this investigation. Inclusion criteria were adults aged 18–40 years who were physically active with no known cardiovascular, renal, or metabolic diseases. Being physically active was defined as completing 75 to 300 minutes of moderate-to-vigorous-intensity activity per week, engaging in resistance exercise ≥2 days per week, or participating in intramural activities (e.g., flag football) (McKay et al., 2022). Exclusion criteria included individuals with diabetes, high cholesterol, or any diagnosed cardiovascular, metabolic, or neuromuscular diseases. Individuals who currently smoke, were pregnant, or had any upper-body musculoskeletal injuries were also excluded from participating in this study. All participants were instructed to abstain from alcohol and vigorous physical activity 24 hours prior to data collection, and from caffeine for 8 hours prior to data collection.

### 2.4 Power analysis

An *a priori* power analysis was conducted using G*Power (v. 3.1.9.7, Düsseldorf, Germany) based on effect sizes reported in previous, relevant investigations (Keller et al., 2023; Landers-Ramos et al., 2026). Because no prior study has evaluated sex-specific changes in NIRS-VOT outcomes in response to exercise, sample size estimation was based on expected main effects of Time and Sex within a mixed-model ANOVA framework, approximating the planned linear mixed-effects models (α = 0.05, power = 0.80). To estimate the Time effect (pre vs. post), an effect size was derived from the change in upslope following submaximal handgrip exercise reported by Landers-Ramos et al., (2026), corresponding to Cohen’s d = 0.499. This value was converted to Cohen’s f = 0.249 for use in the ANOVA-based power analysis, yielding an estimated required sample size of 24 participants. To estimate the Sex effect, baseline (PRE) upslope data from our previous matched ischemic vasodilatory stimulus study (Keller et al., 2023) were used. Mean upslope values were 1.87 ± 0.37%·s^-1^ in men and 1.23 ± 0.59%·s^-1^ in women, corresponding to a large effect size (Cohen’s d = 1.28). Assuming α = 0.05 and power = 0.80, the estimated required sample size was 10 participants per group (20 total participants). Based on these calculations, a total sample size of approximately 20–24 participants was expected to provide adequate power to detect the primary main effects of Time and Sex within the mixed factorial design. However, because no prior studies have quantified sex-specific changes in NIRS-VOT responses following exercise, there were insufficient data to estimate an effect size for the Sex × Condition × Time interaction. Therefore, the study was not specifically powered to detect higher-order interaction effects, and the three-way interaction findings should be considered exploratory and interpreted accordingly. It should also be acknowledged that the analyses performed in the current study used linear mixed-effects models, whereas the *a priori* power analysis was based on mixed-model ANOVA procedures. As such, the power analysis accounted for the repeated-measures design but did not explicitly incorporate participant-level random effects or covariate adjustments.

### 2.5 Measurements

#### 2.5.1 Body composition and ultrasonography

Participants’ total body mass and height were measured using an ultrasonic stadiometer and scale, and bioelectrical impedance analysis (SECA mBCA Ultra, Germany) was used to quantify fat mass, lean body mass, and percent body fat. Subcutaneous ATT was measured using B-mode ultrasonography with the linear distance function (12L-RS; 12 MHz; GE LOGIQ e, GE Healthcare, USA). This value was used in accounting for the influence of ATT on the NIRS signal and to ensure adequate spatial resolution (Barstow, 2019; Keller et al., 2023; Landers-Ramos et al., 2024; Murias et al., 2026).

#### 2.5.2 Exercise protocol

Participants performed 2 maximal voluntary isometric contractions (MVIC) using an isometric handgrip dynamometer (Biometrics Ltd, Newport, UK) that integrated with our data acquisition system (PowerLab, ADInstruments). If a substantial increase (i.e., ≥5%) was observed between attempts, then a third MVIC was performed. All attempts were interspaced by 1 min, and the highest force achieved was recorded. Following this, participants completed 30 rhythmic handgrip contractions at 50% MVIC, with real-time visual feedback at 50 bpm provided by a metronome. Each contraction was performed for two metronome beats followed by two beats of relaxation, corresponding to 2.4 s of contraction and 2.4 s of relaxation per cycle. Thus, the exercise bout lasted ~144 s and used a 50% duty cycle. Although fatigue was not directly quantified following the exercise bout, the protocol was designed to minimize fatigue-related effects. Previous investigations using the same relative load (i.e., 50% MVIC) and intermittent handgrip exercise to task failure reported substantially greater numbers of contractions and longer cumulative contraction times than those used in the present study. Additionally, in the current study, participants were instructed to maintain force output within ±2.5% of 50% MVIC during all contractions; these thresholds were displayed using two visual threshold markers on the force trace. Further, no participant terminated the protocol prematurely or was unable to maintain the prescribed target force throughout the exercise bout.

#### 2.5.3 Near-infrared spectroscopy

The StO_2_ signal was recorded using a continuous wave (750 nm and 850 nm, spectral distribution 60 nm and 80 nm FWHM) NIRS device (moorVMS-NIRS, Moor Instruments Ltd, Axminster, UK). Specifically, prior to data acquisition, the device was calibrated to a standardized calibration block (moorVMS-NIRS-CAL, Moor Instruments Ltd) with reference values for StO_2_ (57.4 – 63.4%) using two photodiode detectors and one source emitter to estimate absolute concentrations of O_2_Hb and HHb (Prahl, 1999). The probe (NP1, Moor Instruments Ltd) was attached parallel to the flexor digitorum superficialis, directly inferior to the medial epicondyle of the humerus, using a black self-adherent wrap. Source-detector separation was fixed at 40 mm using a probe holder mold (NPH1-40, Moor Instruments Ltd). Data were recorded at a sampling frequency of 5 Hz. The StO_2_ data were simultaneously digitized (PowerLab, ADInstruments, Sydney, Australia) with the isometric force data and analyzed in LabChart Pro (v.8).

#### 2.5.4 Vascular occlusion test

The VOT protocol consisted of a 3-minute baseline, arterial occlusion (250 mmHg) until an absolute StO_2_ of 40% was achieved (i.e., standardizing the ischemic vasodilatory stimulus) or a maximum of 5 minutes of occlusion, and a subsequent 3-minute reperfusion period. A rapid inflation/deflation device (E20s, Hokanson Inc., Bellevue, WA, USA) was used to inflate a cuff (SC10D, Hokanson Inc.) around the proximal upper arm. The NIRS-VOT outcomes were derived from the StO_2_ signal (**Figure 1**). Specifically, the rate of desaturation (downslope) was calculated as the linear slope of the StO_2_ signal during the initial phase of ischemia. For trials with ischemic durations greater than 30 s, the first 30 s following cuff inflation were used. When the target StO_2_ threshold (40%) was reached in less than 30 s, downslope was calculated using the entire ischemic period. Further, the cumulative oxygen (O_2_) deficit was calculated as the integral of the StO_2_ signal relative to the baseline value immediately before the onset of occlusion (i.e., area between the occlusion curve and the initial StO_2_ value). To be clear, the duration of ischemia was initially considered; however, the integral measure is determined not solely by occlusion duration but also by the magnitude and temporal profile of the StO_2_ response, thereby providing a more comprehensive representation of the ischemic stimulus beyond StO_2min_ or occlusion time alone. For the resaturation metrics, upslope was defined as the linear slope of the first 10 seconds of reperfusion; StO_2max_ was the highest StO_2_ value observed during reperfusion; and T_1/2_ was defined as the time in seconds (s) for the StO_2_ signal to increase from StO_2min_ to half of StO_2max_ after cuff release. 

**Figure 1:**
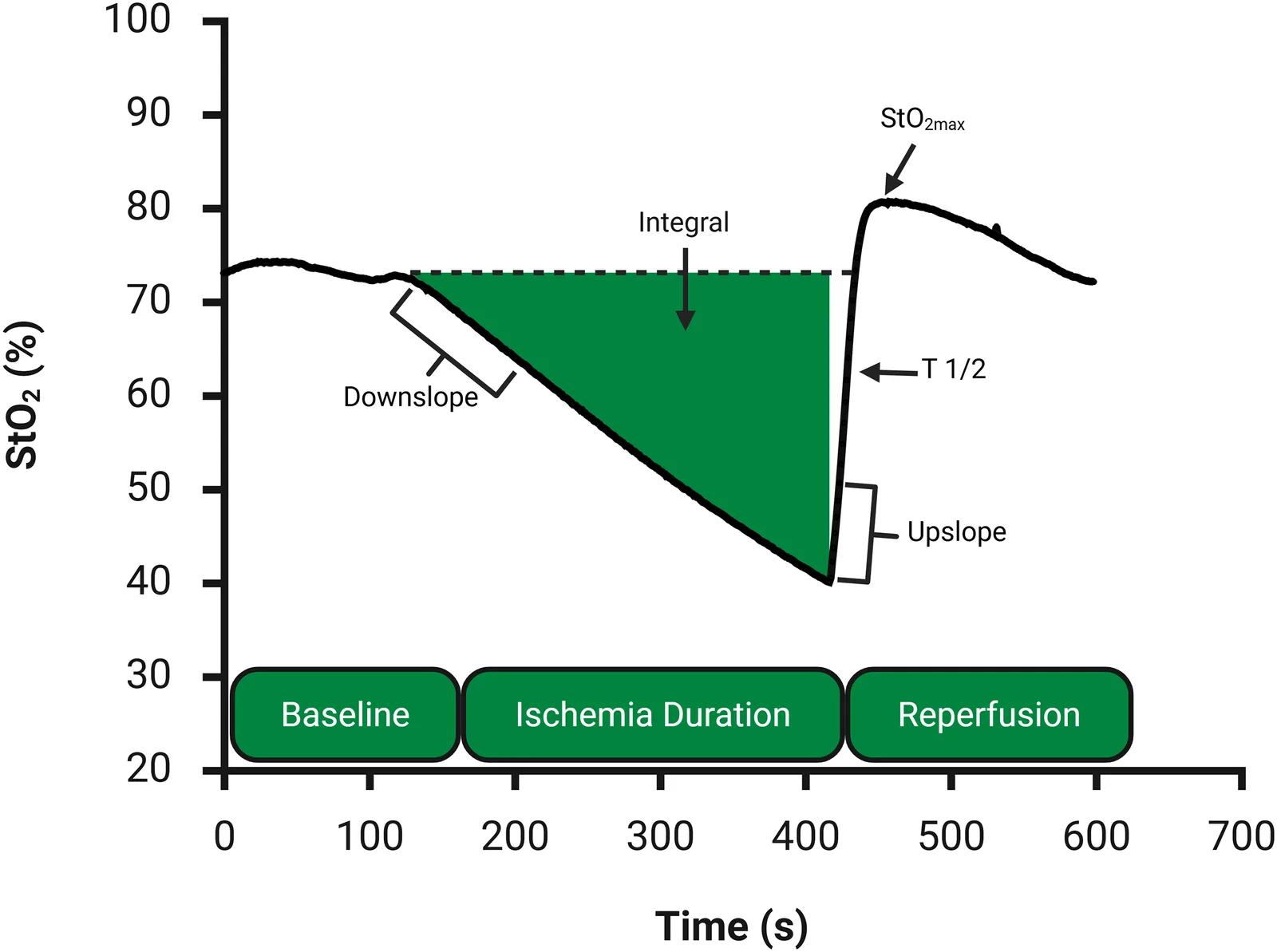
This schematic depicts a representative tracing of the StO_2_ signal during a near-infrared spectroscopy vascular occlusion test (NIRS-VOT) and the relevant outcome variables. Following a baseline period, arterial occlusion was used to induce ischemia, resulting in a progressive decline in skeletal muscle oxygenation (StO_2_). The rate of decline was quantified as the downslope, whereas cumulative O_2_ deficit (integral) was calculated as the area between the baseline StO_2_ value and the ischemic desaturation curve. Upon cuff release, reperfusion resulted in a rapid increase in StO_2_. The initial rate of resaturation was quantified as the upslope, the highest StO_2_ value achieved during reperfusion was defined as StO_2max_, and T_1/2_ was defined as the time required for the StO_2_ signal to increase from StO_2min_ to one-half of StO_2max_.

### 2.6 Statistical analysis

To examine acute changes in NIRS-VOT responses, separate linear mixed-effects models were fitted for NIRS-VOT variables: downslope, upslope, T_1/2_, and StO_2max_. Each model included fixed effects for Sex (MALE, FEMALE), Condition (EX, CON), and Time (PRE, POST), as well as all interaction terms. Adipose tissue thickness was included as a covariate to account for its potential influence on NIRS-derived signals. Participant was included as a random intercept to account for repeated measures, and models were estimated using restricted maximum likelihood. When significant interactions were detected, *post hoc* contrasts of estimated marginal means were performed with Tukey adjustment for multiple comparisons. In the absence of significant interactions, main effects were interpreted using pairwise comparisons of estimated marginal means. To explore potential mechanistic contributors to changes in StO_2max_, an additional mixed-effects model was constructed, including cumulative O_2_ deficit (i.e., integral) as a predictor. This exploratory analysis examined whether variation in cumulative O_2_ deficit was associated with StO_2max_ and whether its inclusion influenced the Condition × Time effect.

In addition, multiple linear regression models were constructed for each resaturation variable (upslope, StO_2max_, and T_1/2_). Pre-exercise values were averaged across conditions to provide a single value per participant. Candidate predictors included sex, ATT, and the integral of the cumulative O_2_ deficit. A model comparison approach was used in which a series of candidate models were specified, including single-predictor, additive, and full models. Model fit was evaluated using Akaike Information Criterion (AIC), Bayesian Information Criterion (BIC), and the coefficient of determination (R²). The most parsimonious model was selected based on lower AIC/BIC values and evidence that additional predictors meaningfully improved model fit. Assumptions of linear regression were assessed using visual inspection of residual and Q–Q plots, as well as formal tests of normality and homoscedasticity. Multicollinearity was evaluated in multivariable models using variance inflation factors. Where deviations from normality were observed, sensitivity analyses (i.e., log-transformation of the dependent variable) were conducted to confirm the robustness of model results. All statistical analyses were performed in R (version 4.6.0) using the *lme4*, *lmerTest*, *emmeans*, and *performance* packages. Data are presented as mean ± SD, and statistical significance was set at α = 0.05.

## 3 Results

*Participant characteristics*. The males were significantly heavier, taller, and had higher body mass index values than the females (all p ≤ 0.002). Age did not differ between the males and females. The females exhibited numerically higher body fat percentage and ATT than the males, but to be clear, these mean differences did not reach traditional statistical significance (**Table 1**).

**Table 2** summarizes the achieved StO_2min_ values and ischemic durations observed during the 50% exercise condition. Although several women reached the maximum occlusion duration before attaining the target threshold, the mean StO_2min_ values remained close to the intended target across sex and time points (39.1% to 42.3%).

**Table 2:** Achieved StO_2min_ and ischemic duration during the PRE and POST NIRS-VOTs of the exercise visit by sex.

Variable	Men		Women	
	Pre	Post	Pre	Post
StO_2min_ (%)	39.1 ± 2.9	40.0 ± 0.0	42.3 ± 4.7	40.9 ± 1.7
Ischemic Duration (s)	165.4 ± 44.7	51.5 ± 39.5	206.3 ± 72.6	97.1 ± 125.1
Trials reaching 300 s (n)	0	0	4	3

Values are mean ± SD unless otherwise indicated; *n* = 12 per sex. PRE and POST refer to assessments performed before and after intermittent isometric handgrip exercise, respectively. Arterial occlusion was terminated when an absolute StO_2_ of 40% was achieved or after the maximum occlusion duration of 300 s. StO_2min_, minimum skeletal muscle tissue oxygenation; NIRS-VOT, near-infrared spectroscopy vascular occlusion test.

### 3.1 Did biological sex contribute to variability in pre-exercise resaturation values (collapsed across condition), and was this contribution retained when considered alongside previously identified covariates and experimental controls?

Mean ± SD values for the pre-exercise NIRS-VOT outcomes, averaged across the exercise and control conditions to provide a single value per participant, are presented in **Table 3**.

**Table 3:** Pre-exercise NIRS-VOT outcomes averaged across the exercise and control visits by sex.

Sex	Upslope (% · s^-1^)	StO_2max_ (%)	T_1/2_ (s)
Males	1.98 ± 0.6	79.9 ± 4.9	14.9 ± 3.9
Females	1.55 ± 0.5	79.0 ± 3.0	15.4 ± 2.3

Values are mean ± SD; *n* = 12 per sex. For each participant, we averaged PRE values from the exercise and control visits to provide one value for each outcome. StO_2max_, maximal skeletal muscle tissue oxygenation during reperfusion; T½, time required for StO_2_ to increase from StO_2min_ to one-half of StO_2max_; NIRS-VOT, near-infrared spectroscopy vascular occlusion test.

*Upslope*. Sex was the strongest predictor of upslope, as the sex-only model demonstrated the lowest AIC (41.32) and BIC (44.85). Models including ATT (AIC = 43.61) or cumulative O_2_ deficit (integral; AIC = 44.50) did not improve model fit relative to the sex-only model. Additionally, adding ATT or integral to the sex model did not improve fit and resulted in higher AIC values (Sex + ATT: AIC = 42.96; Sex + Integral: AIC = 43.19). The full model (Sex + ATT + Integral) further worsened model fit (AIC = 44.91). Sex accounted for approximately 15% of the variance in upslope (R² = 0.15). In the final model, sex exhibited a positive effect on upslope (β = 0.43), indicating higher values in males.

*StO_2max_. Cumulative* O_2_ deficit was the primary predictor of StO_2max_, as this measure substantially improved fit relative to the intercept-only model (AIC = 126.93 vs. 137.55; p < 0.001). The addition of sex further improved model fit (AIC = 124.39; p = 0.049), resulting in the best-fitting model. The model, including ATT and integral, demonstrated lower explanatory power (AIC = 126.00), and ATT was not a significant predictor (p = 0.11), indicating it did not meaningfully contribute to model fit. The final model, including sex and integral, explained approximately 51% of the variance in StO_2max_ (R² = 0.51). Within this model, both cumulative O_2_ deficit (β = 0.0034, p < 0.001) and sex (β = 2.61, p = 0.049) were significant predictors, indicating that sex contributed to variability in StO_2max_ beyond that explained by differences in ischemic stimulus.

*T_1/2_. Cumulative* O_2_ deficit was the sole predictor of T_1/2_, as the integral-only model demonstrated the lowest AIC (121.97) and BIC (125.50). The addition of ATT or sex did not improve model fit (ATT + Integral: AIC = 122.6; Sex + Integral: AIC = 123.8), and ATT was not a significant predictor (p = 0.28). The full model (Sex + ATT + Integral) did not improve overall model fit (AIC = 123.9). The integral-only model explained approximately 23% of the variance in T_1/2_ (R² = 0.23). In this model, cumulative O_2_ deficit was a significant positive predictor (β = 0.0017, p = 0.018), indicating that greater cumulative ischemic stimulus was associated with slower time to StO_2max_. Results were unchanged after log-transformation of T_1/2_ (p = 0.012).

### 3.2 Exploratory analysis of the effects of acute isometric handgrip exercise on NIRS-VOT responses and potential sex-specific differences

Mean and individual PRE and POST responses during the exercise condition are presented for downslope (**Figure 2A**), cumulative O_2_ deficit (**Figure 2B**), upslope (**Figure 2C**), and T_1/2_ (**Figure 2D**). Corresponding descriptive values for the control condition are presented in **Table 4**.

**Figure 2:**
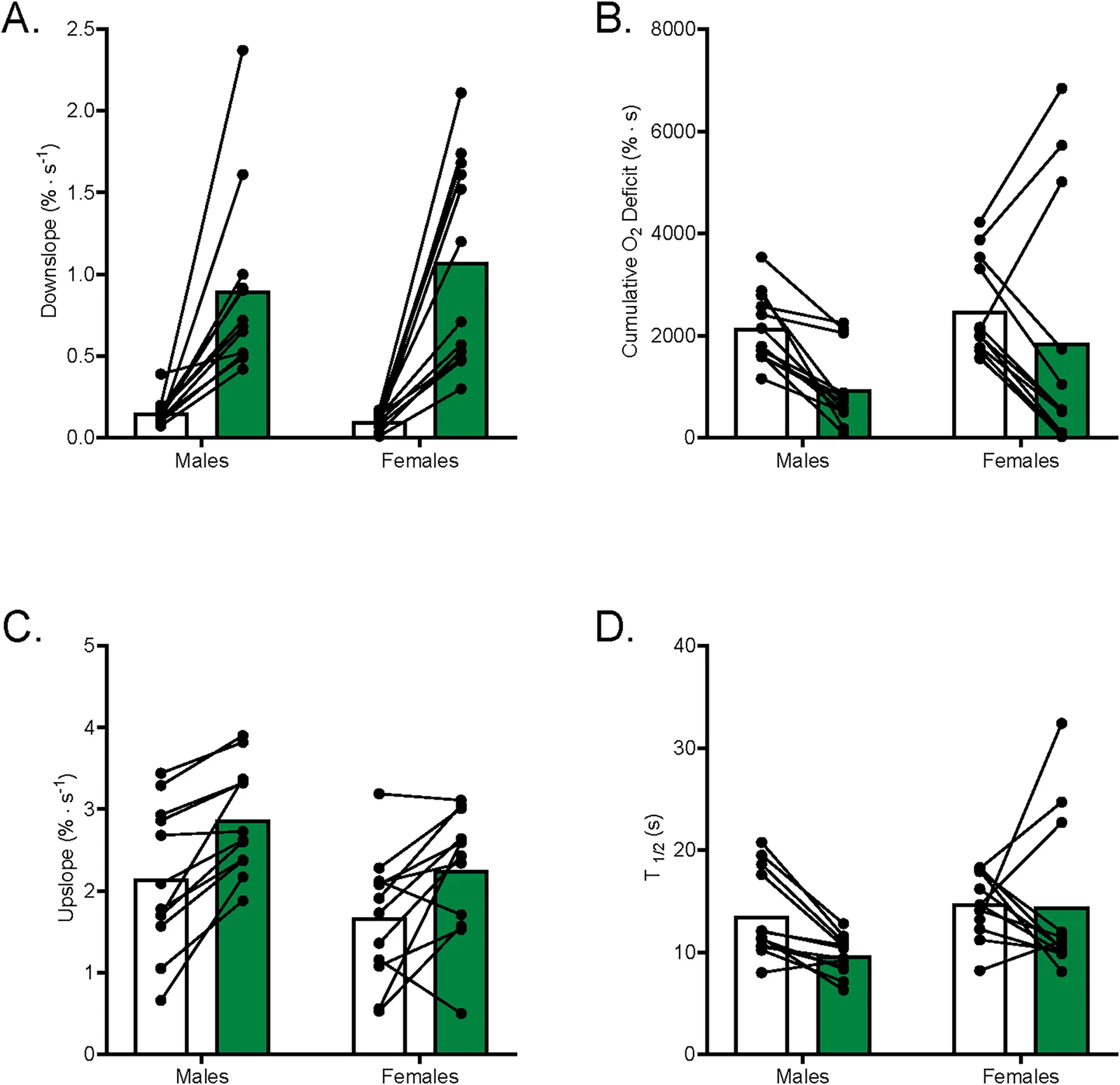
Pre (white bars) and post-handgrip (green bars) mean values with individual values corresponding to near-infrared spectroscopy vascular occlusion test (NIRS-VOT) outcomes in males and females. Panels depict **(A)** downslope (%·s^-^¹), **(B)** cumulative O_2_ deficit (%·s), **(C)** upslope (%·s^-^¹), and **(D)** T_1/2_(s). Individual participant changes are shown with connected lines. Following exercise, downslope and upslope increased, while cumulative O_2_ deficit and T_1/2_ decreased, with no consistent sex-specific effects observed.

**Table 4:** Mean ± standard deviation NIRS-VOT values for the non-exercise control condition.

Variable	Men		Women	
	Pre	Post	Pre	Post
Downslope (% · s^-1^)	0.155 ± 0.06	0.138 ± 0.07	0.133 ± 0.05	0.153 ± 0.15
Cumulative O_2_ deficit (% · s)	2117.6 ± 1059.1	2309.9 ± 992.1	2785.7 ± 1259.3	2617.4 ± 1183.7
Upslope (% · s^-1^)	1.81 ± 0.56	2.20 ± 0.7	1.42 ± 0.77	1.49 ± 0.65
StO_2max_ (%)	80.2 ± 6.3	79.7 ± 7.0	78.7 ± 4.0	79.2 ± 3.9
T_1/2_ (s)	16.3 ± 5.0	14.4 ± 3.8	16.1 ± 4.1	15.7 ± 4.8

Values are mean ± SD; *n* = 12 per sex. PRE and POST refer to assessments performed before and after 5 min of seated rest, respectively. Cumulative O_2_ deficit represents the integral of the difference between baseline StO_2_ and the StO_2_ response during ischemia. StO_2max_, maximal skeletal muscle tissue oxygenation during reperfusion; T½, time required for StO_2_ to increase from StO_2min_ to one-half of StO_2max_; NIRS-VOT, near-infrared spectroscopy vascular occlusion test.

*Downslope.* There was no significant Sex × Condition × Time interaction (F(1, 66) = 0.63, p = 0.43, $\eta_{p}^{2}$ = 0.009); however, a significant Condition × Time interaction was observed (F (1,66) = 49.77, p <.001, $\eta_{p}^{2}$ = 0.43). *Post hoc* comparisons, collapsed across Sex, demonstrated a significant increase in downslope from PRE to POST in the exercise condition (mean difference = 0.86%·s^-1^, p <.001), with no change observed in the control condition (p = .99; **Figure 2A**; **Table 4**). Additionally, there was no main effect of sex (F(1,21) = 1.90, p = .18, $\eta_{p}^{2}$ = 0.08). For this model, ATT was a significant covariate (F(1,21) = 5.54, p = .028, $\eta_{p}^{2}$ = .21), such that greater ATT was associated with slower desaturation rates.

*Upslope.* There was no significant Sex × Condition × Time interaction (F(1,66) = 0.12, p = .73, $\eta_{p}^{2}$ = .002), and similarly, there was no significant Condition × Time interaction (F(1,66) = 2.69, p = .11, $\eta_{p}^{2}$ = .04). There were, however, significant main effects of Sex (F(1,21) = 5.04, p = .036, $\eta_{p}^{2}$ = .19), Condition (F(1,66) = 15.29, p <.001, $\eta_{p}^{2}$ = .19), and Time (F(1,66) = 11.42, p = .001, $\eta_{p}^{2}$ = .15) (**Figure 2C**). *Post hoc* comparisons indicated that upslope was faster in males than females (mean difference = 0.48%·s^-1^, p = .036), faster in the exercise condition compared to control (mean difference = 0.51%·s^-1^, p <.001), and increased from PRE to POST (mean difference = 0.44%·s^-1^, p = .001). For this model, ATT was not a significant covariate (F(1,21) = 0.59, p = .45, $\eta_{p}^{2}$ = .03).

*StO_2max_.* There was not a significant Sex × Condition × Time interaction (F(1,66) = 0.79, p = .38, $\eta_{p}^{2}$ = .01); however, a significant Condition × Time interaction was observed (F(1,66) = 12.42, p <.001, $\eta_{p}^{2}$ = .16). *Post hoc* comparisons, collapsed across Sex, demonstrated a significant decrease in StO_2max_ from PRE to POST in the exercise condition (mean difference = 5.12%, p <.001; **Figure 3A**), with no change observed in the control condition (p = .97). There was no significant main effect of Sex (F(1,21) = 0.57, p = .46, $\eta_{p}^{2}$ = .03), and ATT was not a significant covariate (F(1,21) = 0.21, p = .65, $\eta_{p}^{2}$ = .01). An exploratory analysis revealed that cumulative O_2_ deficit was a significant positive predictor of StO_2max_ (β = 0.0017, p <.001), indicating that a greater deficit was associated with higher StO_2max_ values (**Figure 3B**). Furthermore, the inclusion of this integral value attenuated, but did not eliminate, the Condition × Time effect (p = .012), suggesting that reductions in cumulative O_2_ deficit contribute to decreases in StO_2max_ following exercise.

**Figure 3:**
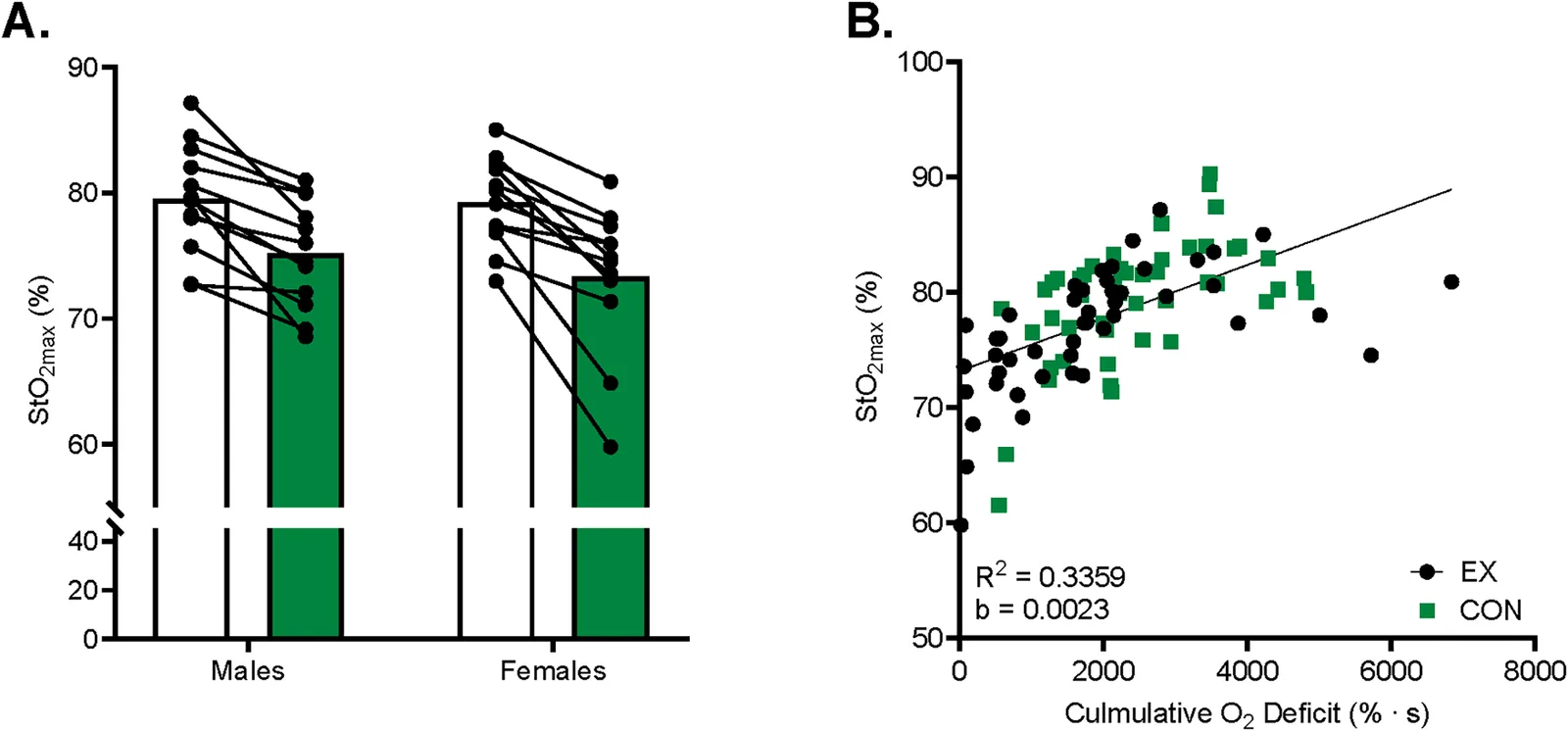
**(A)** Pre (white bars) and post-handgrip (green bars) mean values with individual responses for StO_2max_ (%) in males and females. Individual participant changes are shown with connected lines. **(B)** Relationship between cumulative O_2_ deficit (%·s) and StO_2max_ (%), with data shown for exercise (EX; black circles) and control (CON; green squares) conditions. The solid line represents the linear regression across all observations, indicating a positive association between cumulative O_2_ deficit and StO_2max_.

*T_1/2_*. There was not a significant Sex × Condition × Time interaction (F(1,66) = 0.39, p = .53, $\eta_{p}^{2}$ = .006) nor a significant Condition × Time interaction (F(1,66) = 0.33, p = .57, $\eta_{p}^{2}$ = .005). However, there were significant main effects of Condition (F(1,66) = 9.97, p = .002, $\eta_{p}^{2}$ = .13) and Time (F(1,66) = 4.06, p = .048, $\eta_{p}^{2}$ = .06), but no main effect of Sex (F(1,21) = 1.16, p = .29, $\eta_{p}^{2}$ = .05). *Post hoc* comparisons indicated that T_1/2_ was greater in the control condition compared to exercise (mean difference = 2.54 s, p = .002) and decreased from PRE to POST (mean difference = 1.62 s, p = .048; **Figure 2D**). ATT was not a significant covariate (F(1,21) = 0.20, p = .66, $\eta_{p}^{2}$ = .01).

## 4 Discussion

The objective of the current investigation was a) to determine whether biological sex contributes to variability in NIRS-VOT outcomes when considered alongside previously identified confounding variables, and b) to explore the extent to which acute isometric handgrip exercise influences NIRS-VOT metrics, particularly in a sex-specific manner. When experimentally controlling StO_2min_ (i.e., 40% threshold) and statistically controlling for ATT, our major findings were as follows: 1) Biological sex explained variability in pre-exercise upslope, whereas the cumulative O_2_ deficit emerged as the primary predictor of StO_2max_; 2) Downslope increased following the handgrip task, consistent with an exercise-induced elevation in mV̇O_2_; 3) the rate of resaturation (upslope) differed across condition and time and was greater in males than females; however, there was no evidence that the effect of exercise was modified by sex; and 4) peak saturation (StO_2max_) was reduced following exercise, which was likely prompted by the ~40% decrease in the cumulative O_2_ deficit. In addition, T_1/2_ was reduced following exercise but did not differ by sex, suggesting that this parameter may be relatively insensitive to sex differences when controlling for StO_2min_. These findings indicate that the physiological determinants of NIRS-VOT responses are outcome-specific, with sex contributing to variability in select parameters but not universally across all measures. Below, these findings are discussed in the context of previous, relevant literature.

We originally hypothesized that men would exhibit greater desaturation and resaturation metrics than women; however, the current results only partially supported this hypothesis. Specifically, although the males demonstrated faster rates of resaturation (upslope), there were no observed between-sex differences for downslope, StO_2max_, or T_1/2_. To be clear, these results were derived from linear mixed-effects models with ATT included as a covariate, alongside experimental control of StO_2min_ at 40%. Consistent with our previous study (Perlet et al., 2024), ATT was a significant covariate for desaturation, with greater ATT associated with lower downslopes. Perhaps counterintuitively, ATT does not exert a consistent attenuation or simple linear scaling effect on NIRS-based signals, as scattering and path length increase nonlinearly with tissue thickness. Simply stated, greater ATT shifts the measurement toward a more superficial, less metabolically active tissue (e.g., subcutaneous adipose tissue), effectively smoothing the skeletal muscle-derived signal and attenuating the rate of desaturation. Thus, as ATT increases, it can attenuate not only the signal amplitude but also its rate of change.

Now considering the resaturation metrics and interpreting the model comparison analyses (pre-handgrip exercise data), these results further indicated that sex was the strongest predictor of upslope, explaining ~15% of the variance, whereas cumulative O_2_ deficit was the primary determinant of StO_2max_ and T_1/2_. Including ATT did not improve model fit for these outcomes, suggesting that sex-related differences in resaturation are not attributable solely to ATT. Previous findings from the lower limb do not completely agree with these current results (Chatlaong et al., 2026). In a sample of 45 healthy, young adults, Chatlaong et al. (2026) reported large sex differences in reperfusion responses in the lower limb (e.g., d = 1.30) that were abolished following adjustment for ATT and desaturation (which explained slightly more unique variance than ATT) characteristics (β = −0.01%·s^-^¹, p = 0.951). These investigators also reported strong associations between desaturation and resaturation slope values (r = 0.6–0.8), highlighting the ischemic insult’s role in subsequent resaturation responses. Interestingly, the present findings also contrast with our previous investigation for upslope (Keller et al., 2023), which reported ATT as the primary factor influencing resaturation, explaining approximately 40% of the variance in upslope and outperforming sex as a predictor. However, in this previous study, the ischemic vasodilatory stimulus (i.e., StO_2min_) was not originally controlled for, and subsequent analyses showed that StO_2min_ provided a superior model fit relative to ATT (Keller et al., 2023). To demonstrate, our past analysis indicated that StO_2min_ provided a superior model fit relative to ATT (ΔAIC/BIC = −3.0; Bayes Factor = 4.6), and the addition of StO_2min_ to ATT-based models improved explanatory power (ΔR² = 0.12), whereas inclusion of ATT in StO_2min_-based models yielded only minimal improvement (ΔR² = 0.07).

The current findings, in conjunction with these past reports, point to an apparent indirect influence of ATT on NIRS-VOT responses, perhaps mediated by its effects on desaturation measures, rather than as an actual determinant of microvascular function. Said differently, in the present study, standardizing StO_2min_ to 40% reduced variability in the ischemic stimulus, perhaps attenuating the influence of ATT on reperfusion-related outcomes. It is therefore possible that the effects of ATT on NIRS-VOT responses may be largely indirect, acting through its influence on the magnitude and rate of desaturation rather than directly altering microvascular reactivity. Intuitively, this interpretation aligns with the notion that greater ATT primarily affects the optical properties of NIRS measurements, rather than directly altering underlying physiology under healthy conditions. Taken together, the current findings emphasize that NIRS-VOT outcomes are influenced by the interplay among tissue characteristics, ischemic stimulus, and metabolic demand, rather than a single factor in isolation.

Our second hypothesis was that exercise would augment NIRS-VOT responses in a sex-specific manner when the magnitude of the ischemic vasodilatory stimulus (i.e., StO_2min_) was experimentally controlled. Contrary to this hypothesis, downslope, upslope, T_1/2_, and StO_2max_ did not exhibit a significant 3-way interaction, indicating that here, exercise did not differentially affect males versus females. Given that the present study was not specifically powered to detect these higher-order interactions, these findings should be interpreted as descriptive rather than definitive and require confirmation in larger, adequately powered investigations. However, it appeared that exercise did influence NIRS-VOT responses, most notably downslope, where a significant interaction was observed. In contrast, upslope and T_1/2_ differed across conditions and time points but showed no significant interactions. Taken together, these findings are generally consistent with the concept that exercise alters oxygen extraction and reperfusion kinetics. As detailed above, sex differences were observed; however, they were primarily seen as main effects and were not modified by exercise, suggesting that, under the current conditions, sex differences are independent of the exercise stimulus. In contrast, Parker et al. (2007) have shown that females exhibit greater femoral vascular conductance (i.e., notably distinct from NIRS-derived measures of resaturation) compared with males during incremental exercise, even when normalized to muscle mass. Although these findings may initially appear inconsistent with the present results, key methodological and physiological distinctions likely account for the differences. Vascular conductance measured during exercise reflects upstream hemodynamic responses, including conduit-artery flow and resistance-vessel dilation under conditions of ongoing metabolic demand. The present investigation included NIRS-VOT–derived metrics capturing downstream microvascular O_2_ kinetics following transient ischemia, with the stimulus magnitude experimentally controlled by adjusting StO_2min_. This is meaningfully different from Parker and others (Parker et al., 2007), who normalized vascular conductance to estimated quadriceps muscle mass to account for sex differences in the magnitude of metabolic activity produced during exercise. However, despite this normalization approach, the metabolic stimulus may still differ between sexes due to differences in absolute workload, motor unit recruitment, and fiber-type–dependent metabolic flux. Thus, this previously observed sex difference in vascular conductance may reflect differences in the magnitude or distribution of the metabolic stimulus rather than intrinsic vascular responsiveness per se. Overall, these findings suggest that sex-related differences in vascular function are context-dependent.

Interestingly, as an exploratory consideration, female participants exhibited a faster rate of resaturation following exercise than male participants under pre-exercise conditions. This occurred despite no statistically significant difference in cumulative O_2_ deficit between post-exercise females and pre-exercise males, with StO_2min_ experimentally controlled across conditions. When taken together, females demonstrated approximately 8% higher upslope values than the males (**Figure 2C**), suggesting that metabolic perturbations may meaningfully drive variability in this metric. Said differently, upslope likely reflects, at least in part, the degree of metabolite accumulation (e.g., adenosine, K^+^), which promotes metabolic vasodilation and enhances functional capillary recruitment, thereby reducing microvascular resistance and facilitating more rapid increases in flow and oxygen delivery. This interpretation can be considered alongside the main effect for Sex (discussed above), which indicates that males exhibit greater upslope values. That is, in the context of previous findings (Rasica et al., 2022; Keller et al., 2023; Citherlet et al., 2024; Traylor et al., 2024), extending this notion to conditions in which males and females are both at baseline, males likely exhibit faster resaturation rates due to a greater ability to produce metabolites, in part because of greater muscle mass and possibly differences in fiber type (i.e., males may exhibit greater glycolytic potential) (Green et al., 1984; Haizlip et al., 2015; Nuzzo, 2024).

In support of this, prior work examining the sex-related differences in the rapid onset of vasodilation demonstrates that young adult females exhibit attenuated vasodilatory responses compared with males during relative-intensity contractions; however, these differences were abolished when absolute workload was matched (Hanson et al., 2021). This corroborates the concept that sex differences in vasodilatory responses are primarily driven by differences in the magnitude of the metabolic stimulus, likely reflecting greater absolute force production (e.g., muscle mass) and greater metabolic perturbation (e.g., fiber type) in males. Moreover, experimental models of exercise-induced fatigue indicate that the decline in contractile function per unit increase in intramuscular metabolites does not differ between sexes (Laginestra et al., 2025). Thus, while metabolic sensitivity appears similar, the overall magnitude of metabolic disturbance differs, suggesting a plausible mechanistic link among sex, metabolite production, and initial reperfusion rates. This points to a muscle-centric framework in which the status of skeletal muscle drives microvascular responses, particularly upslope. Future investigations should seek to isolate the contribution of metabolic factors (e.g., via clamped or pharmacologically mediated conditions) to more precisely define their role in sex differences in NIRS-derived resaturation metrics. In addition, further work is needed to clarify the extent to which these measures predict disease risk and to better distinguish whether previously observed associations are attributable more to skeletal muscle properties than to vascular responsiveness (Rosenberry et al., 2019; Rosenberry and Nelson, 2020).

To our surprise, StO_2max_ consistently decreased following exercise (**Figure 3A**). That is, our original assumption was that StO_2max_ would be augmented following exercise, like upslope, given the heightened metabolic load and potential increases in capillary recruitment. However, as in previous reports (Rasica et al., 2022; Keller et al., 2023), the current findings suggest that peak reoxygenation is independent of the rate of resaturation, but may instead be constrained by the magnitude of the preceding ischemic stimulus. This further supports the notion that NIRS-derived microvascular responses reflect, in part, intrinsic skeletal muscle metabolic characteristics rather than vascular function alone. One explanation for this observed reduction is the diminished cumulative O_2_ deficit, largely due to the hastened rate of desaturation (**Figure 2A**). Specifically, the accelerated rate of desaturation (**Figure 2A**) led to a more rapid attainment of the predetermined StO_2min_ threshold (40%), thereby shortening the duration and magnitude of the cumulative O_2_ deficit. This is physiologically meaningful, as peak reactive dilation increases with occlusion duration (seconds), so a shorter ischemic period limits the achievable peak response (Koller and Bagi, 2002; Alvares and Soares, 2024). Mechanistically, this may be explained by reduced activation of mechanosensitive signaling pathways during ischemia, including endothelial deformation, which stimulates nitric oxide (NO) release, thereby contributing to peak vasodilatory responses during reperfusion (Koller and Bagi, 2002). However, this NO-mediated dilation opposes pressure-induced myogenic vasoconstriction. As conjecture, under conditions of a shortened ischemic period (i.e., post-exercise), diminished endothelial deformation may reduce NO bioavailability, thereby allowing myogenic influences to exert a relatively greater constraint on peak reoxygenation. In contrast, flow- and shear-mediated mechanisms primarily influence the rate and duration of the reperfusion response, rather than the peak itself. As such, enhancements in post-exercise blood flow may accelerate reoxygenation kinetics (i.e., upslope), but do not substantially increase the maximal level of reoxygenation achieved. Instead, StO_2max_ appears to be at least partially determined during the ischemic phase, where the magnitude and duration of endothelial deformation govern the subsequent vasodilatory capacity. Consequently, a reduced ischemic stimulus following exercise would limit the attainable StO_2max_, even in the presence of enhanced reperfusion dynamics.

A second, likely non-mutually exclusive, explanation for the decrease in StO_2max_ following exercise, observed in all participants (see individual responses), is the elevated mV̇O_2_ that likely persisted throughout the reperfusion phase. That is, post-exercise reperfusion may reflect a closer coupling between O_2_ delivery and metabolic demand, such that although delivery increased, utilization increased as well. Consequently, unlike the resting condition, in which lower metabolic demand permits a greater overshoot in StO_2_, post-exercise muscle may continue to extract and consume O_2_ as it is delivered at a higher rate. This would effectively blunt the rise in tissue saturation and limit the attainable StO_2max_ despite faster reoxygenation kinetics. Taken together, these findings suggest that StO_2max_ is determined, at least in part, by the dynamic balance between O_2_ delivery and utilization. Further, these proposed explanations should be considered hypotheses that require additional investigation to be established as the precise mechanisms responsible for these responses.

The current study is not without experimental considerations and limitations, and readers should consider the following when evaluating the results and interpretations. First, we did not control for the menstrual cycle or pharmacological contraceptive use. To be clear, females were assessed across phases in a randomized manner; however, past results indicate that these factors exert minimal influence on NIRS-VOT outcomes, with consistently small effect sizes (≤0.10) reported for key metrics such as upslope (Mattu et al., 2020). Accordingly, while hormonal influences cannot be definitively excluded, their impact on the present findings is likely modest. Second, by chance, the present sample did not exhibit a statistically significant sex difference in ATT (**Table 1**), which was unexpected. Of note, measurements were obtained from the forearm, a region that typically has less subcutaneous fat than other commonly studied sites (e.g., the vastus lateralis), potentially reducing the magnitude of observable between-sex differences. Despite the absence of a statistically significant difference, exploratory Bayesian analyses indicated a high probability (~94%) that females exhibited greater ATT than males, although the 95% highest density interval included zero. Thus, our sample appears at least quasi-consistent with past studies reporting baseline sex differences in ATT. Third, we used a less commonly reported NIRS device (i.e., moorVMS-NIRS) in this line of research. Although unlikely, differences in device characteristics across manufacturers may influence measurement outcomes and resulting interpretations. Another limitation is that we did not randomize the exercise and control conditions, as we added the control visit after IRB approval of a protocol amendment. Thus, we cannot completely rule out potential order or familiarization effects, although including the repeated VOT control condition allowed us to partially account for the influence of repeated testing. Further, although the VOT protocol was designed to standardize the ischemic stimulus by targeting a StO_2min_ of 40%, several participants reached the maximum occlusion duration before attaining this threshold. Consequently, although the achieved StO_2min_ values remained relatively close to the intended target, some variability in the ischemic stimulus persisted across participants. Additionally, because these observations reached the prespecified maximum occlusion duration, truncation of the ischemic period may have influenced the calculated cumulative O_2_ deficit for those trials. Moreover, although cumulative O_2_ deficit was included to provide a more comprehensive representation of the ischemic challenge by incorporating both the magnitude and duration of desaturation, it may not fully capture all duration-dependent mechanosensitive, myogenic, or endothelial processes that contribute to subsequent reperfusion responses. Lastly, our modest sample size and single-center recruitment may limit the precision and generalizability of these findings, particularly for higher-order interactions. Although the *a priori* power analysis supported the sample size for the specified main effects of Time and Sex, the study was not powered to detect Sex × Condition × Time interactions. Accordingly, the absence of significant higher-order interactions should not be interpreted as evidence of equivalent responses between males and females. These analyses should be considered exploratory and require confirmation in larger investigations.

In conclusion, here we demonstrated that the factors contributing to variability in NIRS-VOT responses are outcome-specific and influenced by both skeletal muscle metabolic characteristics and microvascular function. Acute isometric handgrip exercise enhanced desaturation and reoxygenation kinetics, yet reduced peak reoxygenation, highlighting a dissociation between the rate and magnitude of reperfusion. A novel finding was that robust analysis indicated that cumulative O_2_ deficit emerged as a primary determinant of StO_2max_, whereas biological sex contributed to variability in upslope, supporting a muscle-centric framework for interpreting these responses. These findings indicate that NIRS-derived measures do not reflect a single aspect of vascular function but rather an integrated response governed by the interaction among metabolic demand, ischemic stimulus, and microvascular function. It is our position that caution is warranted when interpreting these metrics as direct indices of vascular health, as they may also capture intrinsic properties of skeletal muscle. Although we observed no evidence of sex-specific exercise effects under the present experimental conditions, these findings should be interpreted with caution, as the study was not powered to detect higher-order interaction effects. Accordingly, future studies with larger sample sizes are needed to determine whether subtle sex-specific responses to acute isometric handgrip exercise exist. Future work should aim to further isolate these contributing factors to improve the physiological interpretation and clinical utility of NIRS-based assessments.

## Data Availability

The raw data supporting the conclusions of this article will be made available by the authors, without undue reservation.
